# Near-Infrared Spectroscopy as a Rapid Screening Method for the Determination of Total Anthocyanin Content in Sambucus Fructus

**DOI:** 10.3390/s20174983

**Published:** 2020-09-02

**Authors:** Stefan Stuppner, Sophia Mayr, Anel Beganovic, Krzysztof Beć, Justyna Grabska, Urban Aufschnaiter, Magdalena Groeneveld, Matthias Rainer, Thomas Jakschitz, Günther K. Bonn, Christian W. Huck

**Affiliations:** 1 Institute of Analytical Chemistry and Radiochemistry, Leopold-Franzens University Innsbruck, Innrain 80-82, 6020 Innsbruck, Austria; Stefan.Stuppner@uibk.ac.at (S.S.); Sophia.Mayr@uibk.ac.at (S.M.); Anel.Beganovic@uibk.ac.at (A.B.); Krzysztof.Bec@uibk.ac.at (K.B.); Justyna.Grabska@uibk.ac.at (J.G.); u.aufschnaiter@mci4me.at (U.A.); m.groeneveld@mci4me.at (M.G.); Matthias.Rainer@uibk.ac.at (M.R.); Analytische-Radiochemie@uibk.ac.at (G.K.B.); 2 ADSI-Austrian Drug Screening Institute GmbH, Innrain 66A, 6020 Innsbruck, Austria; Thomas.Jakschitz@adsi.ac.at

**Keywords:** NIR spectroscopy, total anthocyanin content (TAC), elderberry

## Abstract

Elderberry (*Sambucus nigra* L., fructus) is a very potent herbal drug, deriving from traditional European medicine (TEM). Ripe elderberries are rich in anthocyanins, flavonols, flavonol esters, flavonol glycosides, lectins, essential oils, unsaturated fatty acids and vitamins. Nevertheless, unripe elderflower fruits contain a certain amount of sambunigrin, a toxic cyanogenic glycoside, whose concentration decreases in the ripening process. Therefore, quality assurance must be carried out. The standard method described in literature is the photometric determination (pH-differential method) of the total anthocyanin content (TAC) that is the highest when the berries are ripe. The drawback of the pH-differential method is the extensive sample preparation and the low accuracy of the method. Therefore, the goal of this publication was to develop a fast non invasive near-infrared (NIR) method for the determination of TAC in whole berries. TAC of elderberries was measured using pH-differentiation method where TAC values of 632.87 mg/kg to 4342.01 mg/kg were measured. Additionally, cyanidin-3-O-glucoside, cyanidin-3-O-sambubioside and cyanidin-3-O-sambubioside-5-O-glucoside which represent more than 98% of TAC in elderberry were quantified using ultra high performance liquid chromatography-multiple wavelength detection—ultra high resolution-quadrupole-time of flight-mass spectrometry (UHPLC-MWD-UHR-Q-TOF-MS) and their sum parameter was determined, ranging between 499.43 mg/kg and 8199.07 mg/kg. Using those two methods as reference, whole elderberries were investigated by NIR spectroscopy with the Büchi NIRFlex N-500 benchtop spectrometer. According to the constructed partial least squares regression (PLSR) models the performance was as follows: a relative standard deviation (RSD_PLSR_) of 13.5% and root mean square error of calibration (RMSECV/RMSEC) of 1.31 for pH-differentiation reference and a RSD_PLSR_ of 12.9% and RMSECV/RMSEC of 1.28 for the HPLC reference method. In this study, we confirm that it is possible to perform a NIR screening for TAC in whole elderberries. Using quantum chemical calculations, we obtained detailed NIR band assignments of the analyzed compounds and interpreted the wavenumber regions established in PLSR models as meaningful for anthocyanin content. The NIR measurement turned out to be a fast and cost-efficient alternative for the determination of TAC compared to pH-differential method and UHPLC-MWD-UHR-Q-TOF-MS. Due to the benefit of no sample preparation and extraction the technology can be considered as sustainable green technology. With the above mentioned inversely proportional ratio of TAC to total amount of toxic cyanogenic glycosides, NIR proves to be a reliable screening method for the ideal harvest time with maximal content of TAC and lowest content of cyanogenic glycosides in elderberry.

## 1. Introduction

Foods that are rich in antioxidants play an essential role in the prevention of cardiovascular and neurodegenerative diseases [1]. Due to the potential nutritional effects, natural antioxidants that are present in food are becoming increasingly interesting. Furthermore, these natural compounds can also be alternatives to synthetic dyes [2]. In *Sambucus nigra* L., fructus (sambucus fructus) the highest concentrated antioxidants are anthocyanins.

Anthocyanins are one of the most common phenolic compounds in fruits and vegetables. As glycosides, they contain glucose, galactose, rhamnose, xylose or arabinose, which are bound to an aglycon. Common anthocyanidins (aglycones) that occur naturally are cyanidin, delphinidin, petunidin, peonidin, pelargonidin and malvidin [3]. Anthocyanins are responsible for the blue, red or purple coloring of fruits and at a wavelength of around 500 nm, the conjugated bonds absorb light and can thus be analyzed using various methods [4]. Epidemiological studies suggest that the consumption of anthocyanins can reduce the risk of cardiovascular diseases, diabetes, arthritis and cancer due to their antioxidative and anti-inflammatory effects [5]. Foods such as juices or jams made from fruits or vegetables that contain anthocyanins are becoming increasingly popular, which likewise elevates the consumption of anthocyanins. Therefore, fruits with a high anthocyanin content, such as berries of the species *Sambucus*, *Lonicera*, *Vitis* and *Viburnum*, are gaining attention [6]. Elderberries are wildly spread in several countries in Europe and are also cultivated on a small scale in some northern European countries [7].

For the detection of anthocyanins, the pH-differential method and different high-performance liquid chromatography (HPLC) methods in combination with a photodiode array detector or mass spectrometry (MS) [7] are most frequently used in the food industry as well as in research [3]. Since HPLC equipment is expensive to purchase, alternative methods are of great interest to many companies and laboratories. The pH-differential method is considered an inexpensive and simple method for the determination of anthocyanins. The method is suitable to determine total monomeric anthocyanin content based on structural changes from flavylium cation at pH 1.0 to carbinol pseudobase pH 4.5. With this method, the TAC can be specified based on a sum parameter. The drawback of the pH-differential method is the extensive sample preparation and the low accuracy of the method. As described by Jungmin Lee et al. the TAC determined by pH-differential method is suitable for authenticity tests of fruit juices but should be used in combination with the quantification of individual anthocyanins using reference methods [8].

Therefore, this study suggests near-infrared (NIR) spectroscopy as a fast, non-invasive and inexpensive alternative. Especially in the pharmaceutical and food industry, NIR spectroscopy has become a widely used technique. Regarding elderberry, NIR spectroscopy has been used to determine the antioxidant capacity of elderflowers [9]. As well as distinguishing elderberry genotypes based on fruit quality [10]. NIR spectroscopy is a very powerful tool for analysis of fruits and vegetables and related food products [11,12]. Our study is the first that describes a NIR spectroscopy technique for the determination of TAC in elderberry.

*Sambucus nigra* is a species within the family of the Caprifoliaceae native to Europe and Asia [13]. There are other species such as *Sambucus adnata* (native to the Himalaya and East Asia), *Sambucus australasica* (native to New Guinea and east Australia), *Sambucus callicarpa* (west coast North America) *or Sambucus ebulus* (Central Southern Europe, Northwest Africa and Southwest Asia),. *Sambucus nigra* occurs as a shrub or small tree that grows up to 6-m in height. Branches contain a pure white marc and the leaf is a compound with up to seven leaflets where six are arranged opposite to each other with one single leaflet at the tip. While the edge of each leaflet is serrated with possible occurring small hairs on the underside, the flowers are flat-topped clusters of tiny, creamy-white flowers with a sweet and summery fragrance. Dried elderflower is traditionally used as sweaty remedy for colds for which there are numerous drugs on the market. Furthermore, many elderberry extracts are sold as food supplements [14]. *Sambucus nigra* is a well described plant, Assessment reports from the European Medicines Agency (EMA) are available for *Sambucus nigra* fructus and *Sambucus nigra* flos [15,16].

*Sambucus fructus* contains several compounds that contribute to pharmacological activity such as large amounts of anthocyanins, mainly cyanidin-3-glucoside and cyanidin-3-sambubioside [17]. Other constituents are flavonols and flavonol esters [17,18,19]. The dried seeds contain lectins, identified as SNA-Ivf [20,21,22], SNA-Vf [21,23,24,25] and Sam n 1 [25]. The fruit contains 0.01% essential oil. Other constituents are organic acids like citric acid, malic acid and viburnic acid [26], as well as vitamins, minerals [26] and carbohydrates like glucose, fructose [26] and pectin [27]. Cyanogenic glycosides were reported by Pogorzelski et al. [24]. Those have been identified in *Sambucus nigra* in fruits, flowers and berries. Cyanogenic glycosides can be found in all edible constituents of the plant. During digestion, the cyanogenic glycosides are hydrolyzed by the enzyme β-glucosidase to produce toxic hydrogen cyanide (HCN) [28]. *Sambucus nigra* folium contains the cyanogenic glycosides zierin, sambunigrin, prunasin and holocalin [29,30]. Maximum residue values between 3 and 17 mg HCN/100 g in leaves and three milligrams HCN/100 g in fruits were defined in the EFSA Compendium of botanicals 2012 [15]. Senica et al. showed a correlation of sambunigrin content and altitude of the plant. With increasing altitude, the content of sambunigrin seems to increase. Values between 28.82 to 209.61 µg/g for *Sambucus nigra* folium, 1.23 to 15.72 µg/g for *Sambucus nigra* flos and 0.11 and 0.59 µg/g for *Sambucus nigra* fructus were measured. Due to the ripening process, sambunigrin decreases and the TAC increases [31]. Therefore, the NIR method was established to provide a tool for rapid screening of degree of ripeness and therefore nontoxicity without sample preparation and extraction in elderberries.

The first step in this study was to collect the elderberries used as a sample set in Tyrol (Austria) at the locations according to Table S1 (Supplementary Materials). After extraction of fresh berries, a new UHPLC-MWD-UHR-TOF-MS method for analysis of TAC was developed. Afterwards the TAC was analyzed with pH-differential method, as it is the industrial standard for determining TAC. NIR measurements of whole elderberries were performed with Büchi NIRFlex N-500 benchtop spectrometer. To construct the PLSR models with the best prediction performance different spectra pretreatments were tried. Quantum chemical simulation was used as a tool for better interpretation of the observed differences. Vibrations in wavenumber regions that are essential for PLSR models were thereby identified. An accompanying workflow chart can be found in the Supplementary Materials (Figure S12).

## 2. Materials and Methods

### 2.1. Chemicals and Samples

LC–MS grade acetonitrile (ACN) was purchased from Fisher Scientific (Hampton, New Hampshire, United States). Gradient grade ethanol (EtOH) was obtained from Merck KGaA (Darmstadt, Germany). Hydrochloric acid 37%, LC-MS grade formic acid (FA) >99.9%,-folded qualitative filter paper 313, 15-mL polypropylene centrifuge tubes with screw cap and PTFE syringe filters (25 mm, 0.45 µm) were obtained from VWR (Radnor, Pennsylvania, United States). Sodium acetate solution 3-M in H_2_O was purchased from Sigma-Aldrich (St. Louis, Missouri, USA). ST-20 Ultraturrax tubes and D6 SiLibeads were purchased from IKA-Werke (Staufen, Germany). cyanidin-3-O-glucoside chloride, cyanidin-3-O-sambubioside chloride and cyanidin-3-O-sambubioside-5-O-glucoside chloride reference standards were purchased from Extrasynthese (Lyon, France). Wild elderberry samples were collected in Tyrol (Austria) at the coordinates presented in Table S1 (Supplementary Materials). Each sample represents the berries attached to one umbel. The umbels were removed and after collection, the wild berries were frozen at minus 20 °C until processing and measurement. Because of the origin of the samples and considerable difficulties in harvesting wild elderberries, the collected sample set was limited to 27 samples (Table S1).

### 2.2. Extraction

One gram fresh elderberry and 5 g extraction solvent (EtOH/FA/H_2_O, 50/5/45, *v:v*:v) were weighed in Ultraturrax tubes and homogenized using glass beads at 6000 rpm for 2 min. Afterwards the Ultraturrax tube was placed in a cooled ultrasonic bath and extracted for 30 min. The extract was filtrated using 0.45-µm funnel filter, 5 g additional extraction solvent were used to ensure a complete transfer of the extract. The extracts were additionally filtrated using a 0.45-µm syringe filter and diluted 1:10 using extraction solvent. The extracts were stored in the dark in falcon tubes (15 mL) at 6 °C.

### 2.3. UHPLC-MWD-UHR-TOF-MS Method

All samples and standard solutions were measured using a UltiMate 3000 System (Thermo Fisher Scientific, Waltham, MA, USA) consisting of an UltiMate 3000 RS Pump, an UltiMate 3000 RS Autosampler, an UltiMate 3000 RS Column Compartment and an UltiMate 3000 RS variable wavelength detector equipped with an Agilent RRHD Zorbax C18 (2.1 × 100 mm, 1.8 µm) column and an Agilent Eclipse Plus C18-Guard column (2.1 × 5 mm, 1.8 µm) (Agilent Technologies, Santa Clara, CA, USA). The mobile phase consisted of ACN (B) and water containing 0.1% FA (A). The HPLC gradient was operated as follows (min/B%): 0/4%, 3/13%, 5/13%, 7/100%, 10/4%, 11/4%. Wavelength detection was set to 279 and 518 nm, injection volume was 1 µL and temperature of the column oven was adjusted to 70 °C.

Limit of detection (LOD) and limit of quantitation (LOQ) were determined from a calibration curve at concentrations ranging from 5 to 40 μg mL^−1^. Calculations of instrumental limits were executed referring to DIN32645:2008–11.

**Figure 1:**
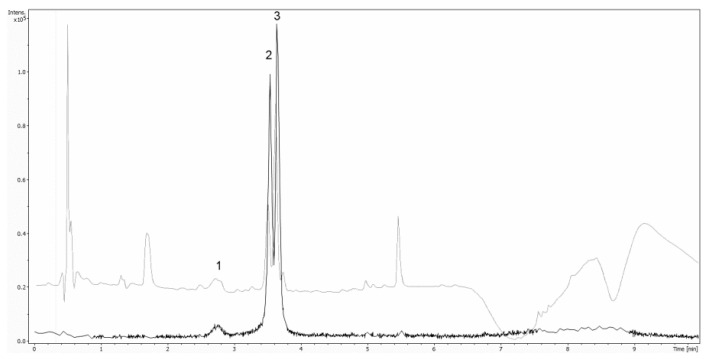
Overlay of extracted ion chromatograms of Cy-3-sam-5-glu (m/z 743.23) (1), Cy-3-glu (m/z 449.12) (2) and Cy-3-sam (m/z 518.17) as well as UV 279 nm.

For MS/MS acquisition and quantification of anthocyanins a Bruker maxis impact UHR-TOF-MS system (Bruker, Bremen, Germany) equipped with an electrospray ionization (ESI) source was used. The system was operated in positive mode and the following settings were applied: end plate offset 500 V, capillary voltage 4500 V, drying gas (N_2_) flow rate, 12.0 L/min; drying gas temperature 120 °C; nebulizer, 3.0 bar, transfer funnel 1 RF 300.0 Vpp, transfer funnel 2 RF 300.0 Vpp, transfer funnel hexapole RF 50.0 Vpp, quadrupole ion energy 5.0 eV, quadrupole low mass 50 m/z, collision cell collision energy 10.0 eV, collision cell collision RF 500.0 Vpp, collision-cell transfer time 50.0 µs, collision-cell -ulse storage 6.0 µs. Auto MS/MS was used, number of precursors was set to 3, absolute threshold was set to 2042 cts and active exclusion after 2 spectra was used. The mass range was set to 80–800 m/z. To guarantee accurate mass in the first 15 s of the HPLC run, a mass calibration using a solution of 250 mL H_2_O, 250 mL 2-propanol, 50 µl FA and 250 mL 1-M NaOH was carried out. The calibration solution was added by an automated calibration delivery system using the loading pump of the UltiMate 3000 System. Figure 1 shows the extracted ion chromatograms of Cy-3-sam-5-glu (m/z 743.23) (1), Cy-3-glu (m/z 449.12) (2) and Cy-3-sam (m/z 518.17) as well as UV 279 nm.

### 2.4. pH-Differential Method

The AOAC pH-differential method describes a photometric method for the determination of total monomeric anthocyanin content in fruit juices, natural colorants and wines within the range of 20–3000 mg/L. The results are presented as cyanidin-3-glucoside equivalents. Total monomeric anthocyanin content is determined by using the absorptivity and molecular weight of cyanidin-3-glucoside, 26,900 ${L\cdot \mathrm{mol}}^{-1}{\cdot \mathrm{cm}}^{-1}$. Due to the usage of 26,900 ${L\cdot \mathrm{mol}}^{-1}{\cdot \mathrm{cm}}^{-1}$ as molar extinction coefficient, TAC values are underestimated compared to HPLC reference analysis. Furthermore, true molar is hard to obtain due to the high hygroscopicity of anthocyanins. It is extremely hard to obtain pure crystalline anthocyanin in adequate quantities in pure crystalline form.

For pH-differential method [8] an Eppendorf BioSpectrometer basic (Hamburg, Germany) was used. Buffer A (0.1-M HCl in H_2_O, pH adjusted with HCl to 1.0) and Buffer B (0.4-M NaOAc in H_2_O, pH adjusted with NaOAc to 4.5) were used. The samples were diluted at a ratio of 1:30 in buffer A and in buffer B, respectively. Absorption (A) was measured at 520 and 700 nm. TAC was determined by Formula 1. MW stands for the molecular weight of cyanidin-3-glucoside (449.2 g/mol), DF represents the dilution factor and ε the molar extinction coefficient (26,900 $\;L\cdot {\mathrm{mol}}^{-1}\;\cdot {\mathrm{cm}}^{-1}$).

(1) $$TAC=\frac{A\cdot MW\cdot DF\cdot 10^{3}}{\varepsilon \cdot 1}$$

### 2.5. NIR Measurements

**Figure 2:**
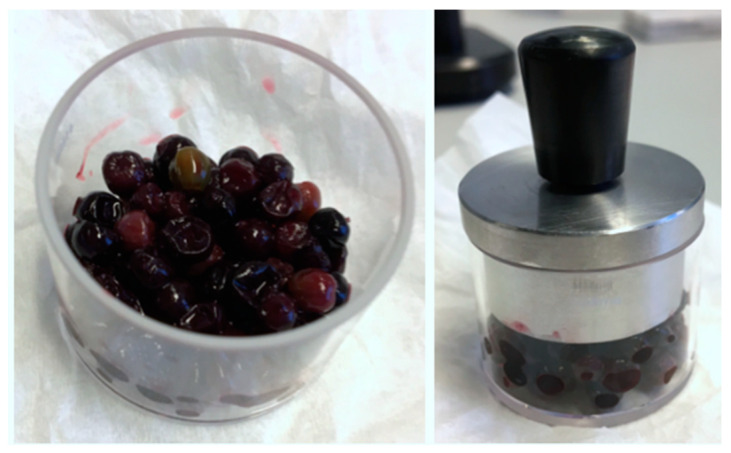
Elderberries compressed for near-infrared (NIR) measurements.

For the NIR measurements, a Büchi NIRFlex N-500 spectrometer (Flawil, Switzerland) equipped with a solids cell attachment was used. The spectra were measured in the range from 10,000 to 4000 cm^−1^ in diffuse reflection mode with a wavenumber accuracy of 2 cm^−1^ and a relative reproducibility of 0.2 cm^−1^. Each sample was measured 9 times with 64 scans with a spectral resolution of 8 cm^−1^ that was automatically interpolated by the software “NIR Ware 1.4.3010” (Büchi, Flawil, Switzerland) to a data point interval of 4 cm^−1^. Technical characteristics of the instrument are highlighted in Table 1. An external Teflon reference was measured after each new sample. The cuvettes used (Hellma GmbH & Co. Kg., Müllheim, Germany) had a technical path length of 2 mm and were made from Quartz SUPRASIL 300 providing transmission of >80% in a spectral range of 50,000 to 4000 cm^−1^ (as indicated by the manufacturer). The samples were unfrozen and measured as soon as they reached room temperature. During measurement, elderberries were compressed using a pressing stamp and a spinner add-on was used for better consistency of measured spectra. (Figure 2).

### 2.6. Method Validation

Validation was completed using the UHPLC-MWD-UHR-TOF-MS method. In this procedure, parameters like linearity, repeatability, method precision and stability of analytes were determined. Linearity was examined using a standard solution of cyanidin-3-O-glucoside, cyanidin-3-O-sambubioside and cyanidin-3-O-sambubioside-5-O-glucoside at concentrations between 25 to 200% of target concentration (25 ppm). Limit of detection (LOD) and limit of quantitation (LOQ) were determined from a calibration curve at concentrations ranging from 5 to 200 mg L^−1^.

Degradation of employed analytes in standard solution was studied was studied and proved to be comparable with reported stability issues of anthocyanins, therefore the standards were prepared on the same day of the analysis and stored in the dark at 4 °C for a maximum of 4 h. Determination of repeatability and the intermediate precision of the pH-differential method was carried out by objecting sample 11 to intra-day (n = 10) and inter-day (n = 30) repeatability measurements (Table S2 in Supplementary Materials).

### 2.7. Spectra Processing and Multivariate Data Analysis

The evaluation of the NIR data was done with the software “The Unscrambler X Version 10.5” (Camo Software, Oslo, Norway). First a transformation from reflectance (*R*) to absorbance (*A*) spectra with a negative common logarithm (log 1/*R*) was applied. Moreover, multiple measurements per sample were reduced to one average spectrum before the following pretreatments were tried to obtain the PLSR models with the highest prediction performance [32,33,34].

The first as well as the second derivative was performed with 5, 7 and 9 smoothing points and a polynomial order 2. An additional SNV-transformation was applied to attempt the reduction of scattering effects. Due to additional wavelength-dependent scattering effects, a detrending transformation and a maximum normalization were attempted for better results as well. For validation, a full cross validation (leave-one-out approach) was used. Additionally, significant wavenumbers were identified with the “uncertainty test” function available in the “The Unscrambler” software [34,35,36].

Relative standard deviation (RSD_PLSR_) and the ratio between the root mean square error of cross validation and root mean square error of calibration (RMSECV/RMSEC) were used as quality parameters for the PLSR model. Formula 2 was used for the calculation of RSD_PLSR_ [35]:

(2) $$RSD_{PLSR}=\frac{RMSECV}{average\;concentration\;range}$$

The closer the value of RMSECV/RMSEC is to 1 and the lower the RSD_PLSR_, the higher the robustness of the quantification model.

### 2.8. Theoretical Simulation of NIR Absorption Bands

Quantum chemical calculations of NIR bands was performed at the second-order vibrational perturbation theory (VPT2) level. The underlying calculations of the electronic structure was based on ONIOM model, in which OH moieties were treated at the density functional theory (DFT) level of theory using B3PLYP density functional with 6–31+G(d,p) basis set. The remaining atoms in the molecular model were subjected to semi-empirical PM6 model of chemistry. This approach enabled efficient anharmonic vibrational analysis while maintaining a reasonable demand for computing resources. Quantum mechanical calculations were carried out with Gaussian 09 Rev.E.03 Software (Gaussian, Inc., Wallingford, CT, USA) [37]. The quantum mechanical study yielded band assignments for the first overtones and binary combination bands, which form the most meaningful contribution to NIR spectra [38].

## 3. Results and Discussion

### 3.1. pH-Differential-Method Measurements

TAC expressed as cyanidin-3-O-glucoside of the elderberry samples are shown in Table 2 and are ranging from 632.87 mg/kg to 4342.01 mg/kg in *Sambucus nigra*. The determined values are in accordance with the ones reported in literature [39,40]. Validation of the method revealed an interday RSD of 2% and an intraday RSD of 10%.

### 3.2. UHPLC-MWD-UHR-TOF-MS Measurements

TAC determination using UHPLC-MWD-UHR-TOF-MS was achieved by quantifying cyanidin-3-O-glucoside (m/z 449.1265), cyanidin-3-O-sambubioside (m/z 581.1736) and cyanidin-3-O-sambubioside-5-O-glucoside (m/z 743.2314).

**Figure 3:**
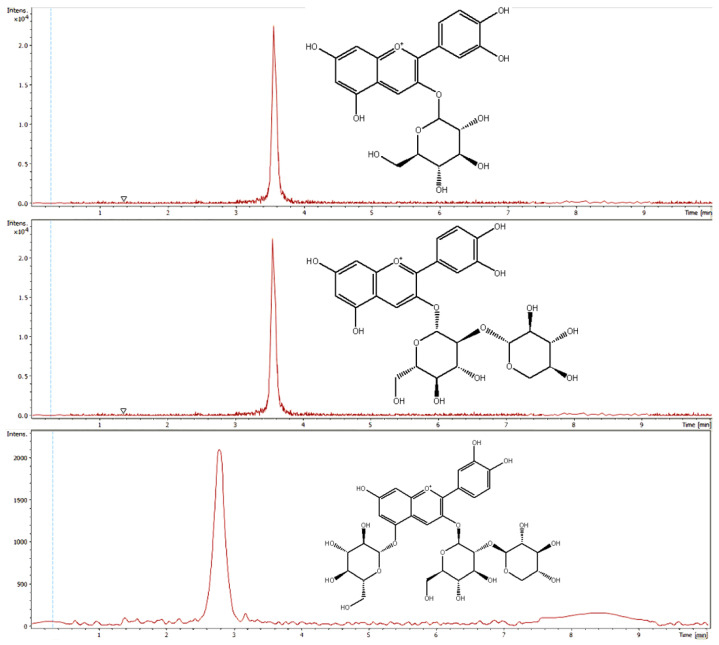
Extracted ion chromatograms of cyanidin-3-O-glucoside, cyanidin-3-O-sambubioside and cyanidin-3-O-sambubioside-5-O-glucoside.

For precise identification of the anthocyanins the aglycone cyanidin (m/z 287.0658) was monitored in MS2. Figure 3 shows the extracted ion chromatograms of cyanidin-3-O-glucoside, cyanidin-3-O-sambubioside and cyanidin-3-O-sambubioside-5-O-glucoside. Cyanidin-3-O-glucoside revealed a coefficient of determination of 0.9923, for cyanidin-3-O-sambubioside the coefficient of determination was ascertained with 0.9913 and cyanidin-3-O-sambubioside-5-O-glucoside showed a R value of 0.9933. For cyanidin-3-O-glucoside a LOD of 3.68 mg/mL and a LOQ of 14.41 mg/mL was determined. Cyanidin-3-O-sambubioside showed a LOD of 3.94 mg/mL and a LOQ of 14.34 mg/mL and for cyanidin-3-O-sambubioside-5-O-glucoside a LOD of 3.302 mg/mL and a LOQ of 12.038 mg/mL was determined.

Contents for each sample and each anthocyanin were determined and values were summed up to obtain the TAC, which was ranging from 499.43 mg/kg to 8199.07 mg/kg. All measurement values are highlighted in Table 3.

Compared to the pH-differential method the UHPLC-MWD-UHR-TOF-MS measurements showed significantly higher values. According to comparison of statistical variance with Fischer’s F-test the two methods are not comparable since test value was higher than critical value.

### 3.3. NIR Spectroscopy

**Figure 4:**
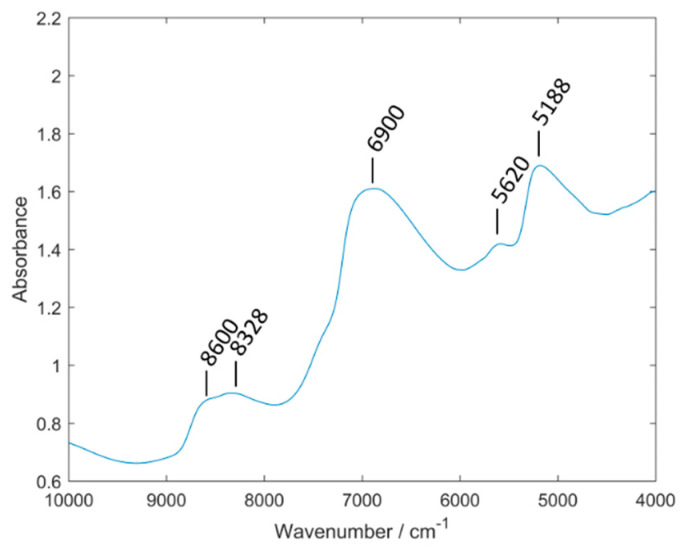
Averaged spectra set of elderberry samples measured with Büchi NIRFlex N-500.

In Figure 4, an averaged spectra set of the elderberry samples measured with the Büchi NIRFlex N-500 can be seen. According to Workman and Weyer the following band assignments were made: 8600 cm^−1^ (C–H stretching second overtone), 8328 cm^−1^ (C–H stretching second overtone), 6900 cm^−1^ (O–H stretching and N–H asymmetric stretching first overtone), 5620 cm^−1^ (C–H symmetric stretching first overtone) and 5188 cm^−1^(O–H stretching and deformation combination) [41]. The complex nature of NIR spectra resulting from extensive overlapping of numerous bands makes it not feasible to identify in the spectrum of an elderberry sample the contributions originating from the absorption bands of the analyzed anthocyanin compounds.

**Figure 5:**
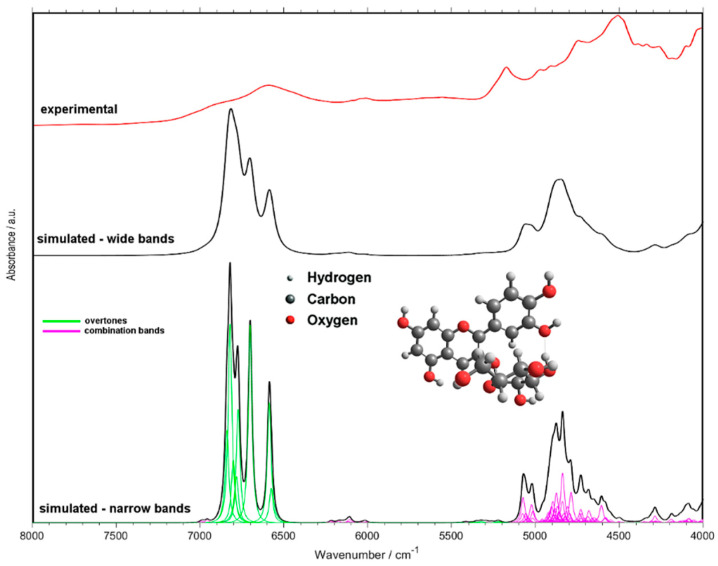
NIR spectrum of cyanidin-3-O-glucoside simulated with use of quantum chemical calculations. Two arbitrary bandwidth levels shown for simulated spectra for the purpose of the discussion. Experimental spectrum was measured for pure standard, polycrystalline cyanidin-3-O-glucoside.

Simulation of NIR spectra using the tools of theoretical chemistry has become feasible in recent years [38]. With aim to interpret the features of the PLSR models established in this study, we performed quantum chemical calculation of the NIR spectra of the three anthocyanins. The simulated spectrum of cyanidin-3-O-glucoside compared with the experimental spectrum of the pure analytical standard (polycrystalline) is presented in Figure 5 while the assignments of the major peaks are listed in Table 4. The figure depicts two theoretical spectra differing in the bandwidth used to model the bands; narrower bands make the spectrum easier for interpretation while upon broadening the spectral line-shape better resembles the experimental one. Additionally, the simulated spectra of cyanidin-3-O-sambubioside and cyanidin-3-O-sambubioside-5-O-glucoside are presented in Supplementary Materials (Figures S10 and S11). As can be noticed, the spectra of the larger molecules do not differ drastically from that simulated for cyanidin-3-O-glucoside, indicating that the similarity of the structural features leads to the similarity of the NIR spectral features. Therefore, further discussion will be based on the simulated spectrum of cyanidin-3-O-glucoside, as the molecule representative for all three substances.

It should be noted, to enable practically feasible simulation of NIR spectra of these molecules, computationally fewer demanding methods were unavoidable (Section 2.8). This has led to minor distortions of the theoretical spectra. Most notably, the spectral intensities of the OH stretching bands (first overtones and binary combinations) are overestimated (Figure 5). It was not unexpected and can easily be accounted for in the discussion, and similar occurrences have been observed by us before [42,43]. Furthermore, the wavenumbers of some of those bands may be underestimated. The molecular models used by us are limited to single molecules, which include the intramolecular hydrogen bonds, however, no intermolecular interactions are described. The typical positions in the case of non-interacting (non-bonded, i.e., not involved in hydrogen bonding) OH groups are ca. 7100–7000 cm^−1^ [43,44,45]. However, one should stress the fact that these inaccuracies have no impact on the subsequent discussion of the features of the PLSR models, as the separation between the selected wavenumber regions is high enough. With this approach, it was possible to unveil which vibrational transitions of anthocyanins are the most meaningful for the analysis of these compounds in elderberry samples using NIR spectroscopy.

In case of Sample S6, S7 and S11 too little elderberries were available, therefore the measured surface was mostly air and the resulting spectra showed errors in absorbance. With both reference data sets, those samples could not be used for the PLSR calculation. For PLSR results with the best prediction performance two completely different approaches for the two sample sets were needed.

As a first pretreatment all spectra were transformed from reflectance into absorbance spectra. In the first case of pH-differential reference method, a detrending transformation with polynomial order 2, a first derivative with 7 smoothing points and a polynomial order 2 as well as a SNV transformation was applied. Sample S10 was identified as an outlier by Hotelling T^2^-test. In the second case, samples correlated with reference analysis from HPLC method needed a maximum normalization as well as a first derivative with 7 smoothing points and a polynomial order 2 to gain the best prediction performance. An additional SNV transformation and a certain wavelength preselection further improved the PLSR prediction performance

**Figure 6:**
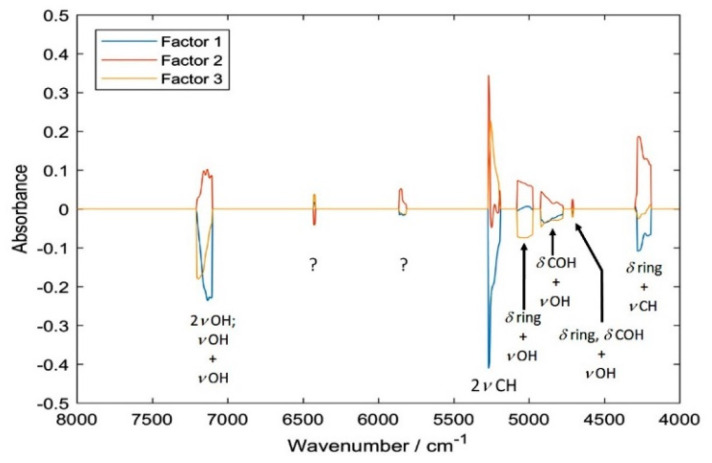
Loadings of the partial least squares regression (PLSR) model correlating NIR spectra of sambucus fructus with UHPLC-MWD-UHR-TOF-MS reference values for TAC.

With the help from the simulated NIR spectrum of cyanidin-3-O-glucoside, an attempt to interpret the structure of PLSR models and to draw conclusions about the matrix effects present in the sample can be made. A comparison of the loadings plot for the PLSR model trained against HPLC reference values presented in Figure 6 (the analogical plot corresponding to the pH-difference reference is presented in Supplementary Materials, Figure S6) with the calculated spectra (Figure 5), leads to the following conclusions. The spectral regions selected as meaningful for the determination of anthocyanin content (Figure 6) can be clearly identified in the spectrum of pure cyanidin-3-O-glucoside (Figure 5). Despite water being a major constituent in fresh elderberry fruits, it seems that unlikely that water bands obscured the signals from anthocyanins and the latter ones could be successfully correlated with the TAC. The two regions meaningful in PLSR model that could potentially be affected by the water signals (just below 7000 cm^−1^ and at ca. 5100 cm^−1^), are located at sufficiently higher wavenumbers and are enough separated from these possible influences. Furthermore, through comparing the PLSR loadings with the experimental spectrum of pure cyanidin-3-O-glucoside, it may be observed that 2*ν*OH and *ν*OH+*ν*OH bands are located at a higher wavenumbers in the case of the former. These bands are very sensitive markers of intermolecular interactions and undergo visible redshift upon formation of hydrogen bonding [46]. This suggests that the anthocyanins on average interact less with the matrix molecules in the fresh fruit that they do in pure polycrystalline form. This should be anticipated, nevertheless, the observation may be helpful for future studies of less evident cases and should be mentioned here. Interestingly, the interpretation of the PLSR loading suggests that anthocyanin content, despite being rich with OH groups in their structures, tends to be well correlated with the vibrations of CH groups (Figure 6 and Table 4). This remains consistent with our previous findings [43]. The structure of the PLSR loadings is comparable between the regressions performed against the reference values known from HPLC (Figure 6) and pH-difference methods (Figure S6); only minor discrepancies may be found there. Explained variance plots and prediction vs reference plots for NIR data with both reference methods can be found in the Supplementary Materials (Figures S4 and S5; Figures S8 and S9).

Summarizing, the following spectral regions identified as meaningful in PLSR models (i.e., leading to the highest accuracy of prediction) were assigned to the corresponding vibrations of anthocyanins. For the regression against the reference values acquired through HPLC: 4188–4516 [(*δ*_ring_, *δ*COH) + *ν*CH), *δ*_ring_ + *ν*CH, *δ*CH + *ν*OH], 4704–4716 (*δ*_ring_, *δ*COH + *ν*OH), 4772–4924 (*δ*COH + *ν*OH), 4976–5076 (*δ*_ring_ + *ν*OH), 5188–5276 (2*ν*CH), 5812–5900 (uncertain), 6416–6432 (2*ν*OH), 7100–7208 (*ν*OH + *ν*OH) and 8956–8968 cm^−1^ (second overtones and ternary combination bands).

On the other hand, the analogical data corresponding to the regression against reference values known from pH-difference method was: 4256–4296 (*δ*_ring_ + *ν*CH); 4336–4408 (*δ*CH + *ν*OH); 4972–5160 (*δ*_ring_ + *ν*OH); 5204–5344 [2*v*CH, (*ν*_as_CH_2_, *ν*CH) + *ν*_s_CH_2_, 2(*ν*_s_CH_2_, *ν*CH)]; 7072–7216 (2*ν*OH and *ν*OH + *ν*OH) and two regions populated by weaker second overtones and ternary combination bands; 8712–8752 and 8792–8804 cm^−1^. These vibrational transitions of anthocyanins can be concluded to be the most meaningful for the quantitative analysis of their content in elderberry samples by NIR spectroscopy.

The best PLSR results for pH-differentiation gained a RSD_PLSR_ of 13.5% and a corresponding RMSECV/RMSEC ration of 1.31. For the HPLC method a RSD_PLSR_ of 12.9% and a corresponding RMSECV/RMSEC ration of 1.28 were achieved. Results of the NIR measurements are summarized in Table 5. NIR spectroscopy provides a rapid screening method for TAC in whole elderberries. NIRS can be calibrated either using pH-differential method, which is widely used in routine applications or for more accurate results the more sophisticated UHPLC-MWD-UHR-TOF-MS method can be used as reference method, providing more accurate results.

The AOAC pH-differential method [8], applicable to monomeric anthocyanin determination, expressed as cyanidin-3-glucoside, was used as one of the two reference approaches. Our resulting TAC values ranging from 632.87 mg/kg to 4342.01 mg/kg are consistent with existing literature [39,40]. The differences of the values is due to their different degree of ripeness of the collected elderberry samples. Nevertheless, the reported results only represent the TAC expressed as cyanidin-3-O-glucoside based on Formula 1. Therefore, compared to the summed up quantity of cyanidin-3-O-glucoside, cyanidin-3-O-sambubioside and cyanidin-3-O-sambubioside-5-O-glucoside quantified using UHPLC-MWD-UHR-TOF-MS lower TAC values are observed. Previous studies also confirmed this behavior. Due to the usage of 26,900 ${L\cdot \mathrm{mol}}^{-1}{\cdot \mathrm{cm}}^{-1}$ as molar extinction coefficient, TAC values are underestimated compared to HPLC reference analysis [41]. Summarizing HPLC is a more accurate reference method, since it describes the actual total anthocyanin content of over 98% of total anthocyanins in *Sambucus nigra*.

The overall TAC determined by UHPLC-MWD-UHR-TOF-MS is ranging between 499.43 mg/kg to 8199.07 mg/kg in the different elderberry samples. These values are also consistent with the described values in the assessment report of the European Union (EMA/HMPC/44208/2012) [15]. The comparison of statistical variance using Fischer’s F-test showed that the methods are not comparable, since test value was higher than critical value. Nevertheless, measurement results showed the same trend in the sample group. Furthermore, both methods were tested for repeatability and intermediate precision. Due to known stability issues of anthocyanins, standards for HPLC measurements were prepared on the same day and used in a time frame of four hours. The pH-differential method showed an interday RSD of 2% and an intraday RSD of 10% which is acceptable for a photometric method.

NIR spectroscopy was observed to be a fast, noninvasive and cost-efficient alternative for screening TAC compared to pH-differential and UHPLC-MWD-UHR-TOF-MS methods. A prediction of TAC was possible with both sample sets, although as already observed with reference data, no comparison of statistical variance was possible. Adapted to respective requirements either pH-differential method or UHPLC-MWD-UHR-TOF-MS is a possible reference analysis for NIR spectroscopy, though MS gives more precise results. By using NIR spectroscopy, wet chemical methods or labor-intensive extractions and other sample preparations are eliminated. The measuring time is drastically shortened, and the measurements can also be carried out without much prior knowledge. Furthermore, new technologies in NIR miniaturization would further enable TAC and therefore nontoxicity screenings directly at the place of cultivation.

## 4. Conclusions

In this study, we confirm NIR spectroscopy as a screening method for TAC in whole elderberries with pH-differential method as well as the more sophisticated UHPLC-MWD-UHR-TOF-MS method used as reference analysis. Due to the benefit of no sample preparation, e.g., extractions with much solvent solution, NIR spectroscopy can be considered as sustainable green technology. Through the analysis of the structures of the constructed PLSR models and with help from quantum mechanically simulated NIR spectra, the impact of the matrix effects with particular attention given to moisture was assessed. NIR spectroscopy presents a reliable method to determine the ideal harvest time with maximal content of beneficial anthocyanins. The concern for the presence of toxic low cyanogenic glycoside content for elderberries remains, due to their low concentrations of 50 mg of cyanide/100 g of fresh fruit, cannot be determined directly with NIR spectroscopy. Therefore, currently an additional measurement of cyanogenic glycosides zierin, sambunigrin, prunasin and holocalin is recommended. However, the essential feature of the biochemistry of the investigated samples [17,19,47,48], the well-known inverse proportionality between TAC and the toxic cyanogenic glycosides content, could become practically useful in the future. With NIR spectroscopy being capable of quantifying TAC in fresh berries, a promising outlook appears for developing a method for determination of the cyanogenic glycosides content indirectly, through analysis of TAC.

## Figures and Tables

**Table 1:** Technical characteristics of Büchi NIRFlex N-500 spectrometer.

Spectral Range	12,500–4000 cm^−1^
Resolution	8 cm^−1^
Spectra per sample	9
Scans per sample	64
Data points	1501
Light source	Tungsten-halogen lamp
Laser type	12 VDC HeNe, up to 633 nm
Monochromator/ Wavelength selection technique	FT-NIR-polarization interferometer with TeO_2_ wedges
Detector	InGaAs
Measuring mode	Diffuse reflection
Dimensions	350 × 250 × 450 mm (w × h × d)
Measuring cell	NIRFlex solids with spinner add-on

**Table 2:** Total anthocyanin content determined by pH-differentiation method.

Sample	Total Anthocyanin Content Represented as Cy-3-glu (mg/kg)	RSD (%)
S1	2428.68	18.5
S3	3885.30	12.9
S6	1716.63	26.8
S7	3121.65	33.9
S9	3264.08	30.1
S10	1191.33	19.2
S11	4342.01	23.7
S12	898.39	24.1
S13	2034.47	12.9
S14	1190.72	18.9
S15	2879.10	17.4
S16	1452.55	28.3
S17	3443.98	33.4
S18	1820.62	33.1
S19	1089.76	21.2
S20	876.83	23.7
S21	632.87	24.1
S22	1330.89	12.9
S23	1132.08	11.5
S24	656.74	12.9
S25	624.12	11.5
S26	1099.06	12.7
S27	1218.08	12.2
S28	1447.48	4.2
S29	1380.31	20.0
S30	1908.79	15.5
S31	1851.28	11.9
R1	168.10	25.8
R2	121.07	33.9
R3	235.73	30.1
R4	276.64	13.2
R5	172.00	20.7
R6	206.23	25.1
R7	240.74	17.9

**Table 3:** Total anthocyanin content determined by UHPLC-MWD-UHR-TOF-MS measurements.

Sample	Total Anthocyanin Content (mg/kg)	RSD (%)
S1	4491.57	2
S3	6866.07	4
S6	2421.38	3
S7	5718.50	5
S9	5563.06	6
S10	2388.63	4
S11	8311.39	3
S12	1154.70	6
S13	2824.30	5
S14	1560.03	1
S15	4277.62	3
S16	2136.57	6
S17	4211.50	5
S18	2644.24	5
S19	1366.76	4
S20	1196.72	5
S21	823.35	1
S22	1710.49	2
S23	1399.16	3
S24	783.02	1
S25	499.43	6
S26	997.69	5
S27	674.17	2
S28	1025.82	1
S29	1581.02	1
S30	1289.57	2
S31	776.00	4
R1	<LOQ	
R2	<LOQ	
R3	<LOQ	
R4	<LOQ	
R5	<LOQ	
R6	<LOQ	
R7	<LOQ	

**Table 4:** Assignments of NIR bands of cyanidin-3-O-glucoside based on quantum chemical calculations.

Wavenumber [cm^—1^]	Assignment ^(a)^
6990.7	*ν*OH + *ν*OH
6958.1	*ν*OH + *ν*OH
6840.3	2*ν*OH
6802.7	2*ν*OH
6821.4	2*ν*OH
6782.7	2*ν*OH
6773.5	2*ν*OH
6701.6	2*ν*OH
6588.1	2*ν*OH
6576.4	2*ν*OH
6219.2	*ν*CH + *ν*OH
6173.8	*ν*CH + *ν*OH
6108.6	*ν*CH + *ν*OH
6013.0	(*ν*_as_CH_2_, *ν*CH) + *ν*OH
5414.1	*ν*CH + *ν*CH
5324.9	2(*ν*_s_CH_2_, *ν*CH)
5289.9	(*ν*_as_CH_2_, *ν*CH) + *ν*_s_CH_2_
5220.8	2*ν*CH
5074.2	*δ*_ring_ + *ν*OH
5024.2	*δ*_ring_ + *ν*OH
4896.3	*δ*COH + *ν*OH
4875.2	*δ*COH + *ν*OH
4839.0	*δ*COH + *ν*OH
4787.7	*δ*COH + *ν*OH
4729.9	*δ*_ring_, *δ*COH + *ν*OH
4680.9	*δ*_ring_, *δ*COH + *ν*OH
4650.1	*δ*_ring_ + *ν*OH
4606.8	*δ*_ring_ + *ν*OH
4585.3	*δ*CH + *ν*OH
4495.7	*δ*CH + *ν*OH
4287.6	*δ*_ring_ + *ν*CH
4190.1	(*δ*_ring_, *δ*COH) + *ν*CH
4084.3	(*δ*_ring_, *δ*COH) + *ν*CH

^(a)^ Notation used: “2” denotes first overtones; “+” sign denotes combination transitions; *ν*—stretching mode; *δ*—deformation mode; as—antisymmetric; s*—*symmetric (mode).

**Table 5:** Parameters of the established partial least squares regression PLSR models.

Reference Analysis	pH Differentiation	HPLC
Samples	27	27
Outliers	3	3
Factors	3	3
R^2^_cal_	0.967	0.979
R^2^_val_	0.927	0.909
RMSECV/RMSEC	1.31	1.28
RSD_PLSR_	13.5%	12.9%
Calibration range	121–4342 *	499–8311 **

* TAC mg/kg expressed as cyanidin-3-O-glycoside, ** summed-up concentration of mg/kg of cyanidin-3-O-glucoside, cyanidin-3-O-sambubioside and cyanidin-3-O-sambubioside-5-O-glucoside.

## Supplementary Materials

The following are available online at https://www.mdpi.com/1424-8220/20/17/4983/s1, Table S1: Latitude and Longitude of the Elderberry collection. Table S2. Validation of pH differential method. Table S3. Band assigments for cyanidin-3-O-sambubioside (compare with Figure S10). Table S4. Band assigments for cyanidin-3-Osambubioside-5-glucoside (compare with Figure S11). Figure S1. Calibration curve for the reference analysis (UHPLC-MWD-UHR-TOF-MS) of cyanidin-3-O-glucoside. Figure S2. Calibration curve for the reference analysis (UHPLC-MWD-UHR-TOF-MS) of cyanidin-3-O-sambubioside. Figure S3. Calibration curve for the reference analysis (UHPLC-MWD-UHR-TOF-MS) of cyanidin-3-Osambubioside-5-glucoside. Figure S4. Prediction vs reference plot of NIR data with pH-differential reference method. Figure S5. Prediction vs reference plot of NIR data with UHPLC-MWD-UHR-TOF-MS reference method. Figure S6. The loadings of the PLSR model correlating NIR spectra of sambucus fructus with pH-differential reference values for TAC content. Figure S7. The loadings of the PLSR model correlating NIR spectra of sambucus fructus with UHPLC-MWD-UHR-TOF-MS reference values for TAC content. Figure S8. Cumulative explained variance plot of the PLSR model correlating NIR spectra of sambucus fructus with pH-differential reference values for TAC content. Figure S9. Cumulative explained variance plot of the PLSR model correlating NIR spectra of sambucus fructus with UHPLC-MWD-UHR-TOF-MS reference values for TAC content. Figure S10. NIR spectrum of cyanidin-3-O-sambubioside simulated with use of quantum chemical calculations. Individual contributing overtone and combination bands are presented. Figure S11. NIR spectrum of cyanidin-3-O-sambubioside-5-O-glucoside simulated with use of quantum chemical calculations. Summed contribution from either overtone or combination bands are presented. Figure S12. Workflow of the research.

## Abbreviations

TAC	Total anthocyanin content
HPLC	High performance liquid chromatography
MS	Mass spectrometry
HCN	Hydrogen cyanide
UHPLC	Ultra high performance liquid chromatography
MWD	Multiple wavelength detector
TOF	Time of flight
NIR	Near-infrared reflectance
RSD	Relative standard deviation
PLSR	Partial least squares regression
RMSECV	Root mean squared error cross validation
RMSEC	Root mean squared error calibration
SNV	Standard normal variation
