# Supplementary material for: Near-Infrared Spectroscopy as a Rapid Screening Method for the Determination of Total Anthocyanin Content in Sambucus Fructus
Source: Sensors (Basel). 2020 Sep 2;20(17):4983. doi: 10.3390/s20174983 (PMC7506738; doi:10.3390/s20174983)
Supplement: Supplementary file 1 [file sensors-20-04983-s001.pdf]

sensors

MDPI

Near infrared spectroscopy as a rapid screening method for the determination of total anthocyanin content in sambucus fructus

Supplementary Materials

Stefan Stuppner[1,2], Sophia Mayr[1], Anel Beganovic[1], Krzysztof Beć[1], Justyna Grabska[1], Urban Aufschnaiter[1], Magdalena Groeneveld[1], Matthias Rainer[1], Thomas Jakschitz[2], Günther K. Bonn[1,2] and Christian W. Huck[1,*]

[1] Institute of Analytical Chemistry and Radiochemistry, Leopold-Franzens University Innsbruck, Innrain 80-82, 6020 Innsbruck, Austria

[2] ADSI-Austrian Drug Screening Institute GmbH, Innrain 66A, 6020 Innsbruck, Austria

* Correspondence: Christian.W.Huck@uibk.ac.at; Tel.: +43 512 507 57304

Table S1. Latitude and Longitude of the Elderberry collection.

Sample description	degree of longitude / °O	degree of latitude / °E
S1	11°22'30,35028"	47°15'48,42936"
S3	11°21'2984544"	47°15'20,46024"
S6	11°22'33,681"	47°15'26,35812"
S7	11°21'57,66696"	47°15'38,7522"
S9	11°22'456456"	47°15'30,924"
S10	11°22'12,5202"	47°16'24,58056"
S11	11°22''12,85788"	47°16'27,5994"
S12	11°22'12,1188"	47°16'21,8442"
S13	11°22'12522"	47°16'23,52216"
S14	11°21'57,21372"	47°16'15,58992"
S15	11°21'32,110548"	47°16'21,20982"
S16	11°23'51,4186"	47°16'16,05612"
S17	11°25'10,6356"	47°15'40,2264"
S18	11°21'26,369"	47°15'20,47"
S19	11°22'33,344"	47°15'26,654"
S20	11°21'58,946"	47°15'13,727"
S21	11°21'40,273"	47°15'17,05"
S22	11°15'0,651"	47°13'47,658"
S23	11°19'41,30124"	47°14'50,91936"
S24	11°18'57,42804"	47°14'49,97796"
R1	10°92′35,651′′	47°22′27,558′′
R2	10°92′35,651′′	47°22′27,558′′
R3	10°92′35,16′′	47°22′33,04′′
R4	10°92′35,16′′	47°22′33,04′′
R5	10°92′29,036′′	47°22′32,809′′
R6	10°92′29,036	47°22′32,809′′
R7	10°92′29,036′′	47°22′32,809′′

Table S2. Validation of pH differential method.

	**Day 1**	**Day 2**	**Day 3**
1	2638.59	2266.87	2008.88
2	2696.87	2275.22	2054.8
3	2661.8	2274.39	1985.5
4	2621.72	2213.44	2020.57
5	2600.02	2244.33	2064.82
6	2575.8	2342.02	2160.84
7	2622.56	2311.96	2090.7
8	2655.96	2349.53	2050.62
9	2598.35	2347.86	2097.38
10	2712.73	2299.44	2176.7
Mean	2638.34	2292.51	2071.08
Standarddeviation	44	46	62
RSD	2%	2%	3%

Table S3. Band assigments for cyanidin-3-O-sambubioside (compare with Fig. S10).

**Wavenumber [cm^{-1}]**	**Assignment** [a)]
6961.885	2🐦OH
6846.014	2🐦OH
6861.453	2🐦OH
6785.449	2🐦OH
6757.406	2🐦OH
6717.978	2🐦OH
6558.512	2🐦OH
6505.660	2🐦OH
6286.199	2🐦OH
6270.684	2🐦OH
6195.627	🐦CH + 🐦OH
6005.357	🐦CH + 🐦OH
5904.773	🐦CH + 🐦OH
5275.323	2🐦CH
5096.103	(🐦COH, 🐦$_{ring}$) + 🐦OH
5083.253	(🐦COH, 🐦$_{ring}$) + 🐦OH
5058.789	(🐦COH, 🐦$_{ring}$) + 🐦OH
5010.365	(🐦COH, 🐦$_{ring}$) + 🐦OH
4694.44	(🐦COH, 🐦$_{ring}$) + 🐦OH
4753.352	(🐦COH, 🐦$_{ring}$) + 🐦OH
4813.062	🐦COH + 🐦OH
4892.365	(🐦COH, 🐦$_{ring}$) + 🐦OH
4553.383	(🐦COH, 🐦$_{ring}$) + 🐦OH
4508.411	🐦$_{wagg}$$CH_2$ + 🐦OH
4471.086	🐦$_{ring}$ + 🐦OH
4293.658	🐦$_{ring}$ +🐦CH

4165.276	ν_{oop}COH + νOH
4064.083	νCOH + νCH
4039.047	ν_{ring} + νCH

[(a)] Notation used: "2" denotes first overtones; "+" sign denotes combination transitions;

ν - stretching mode; ν - deformation mode; as – antisymmetric;

s – symmetric (mode).

Table S4. Band assigments for cyanidin-3-Osambubioside-5-glucoside (compare with Fig. S11).

**Wavenumber [cm⁻¹]**	**Assignment** [a)]
6885	2νOH
6860	2νOH
6795	2νOH
6780	2νOH
6680	2νOH
6645	2νOH
6609	2νOH
6568	2νOH
6318	2νOH
6227	νCH +νOH
6166	νCH +νOH
6114	◎νCH,$\nu_s$$CH_2$]+$\nu$OH
6063	◎νCH,$\nu_{as}$$CH_2$]+$\nu$OH
6024	◎νCH,$\nu_{as}$$CH_2$]+$\nu$OH
5933	◎νCH,$\nu_{as}$$CH_2$]+$\nu$OH
5905	◎νCH,$\nu_{as}$$CH_2$]+$\nu$OH
5411	2νCH
5325	◎νCH,$\nu_s$$CH_2$]+◎◎$\nu$CH,$\nu_s$$CH_2$]
5278	◎νCH,$\nu_{as}$$CH_2$]+$\nu_s$$CH_2$
5235	2νCH, 2$\nu_s$$CH_2$
5105	◎◎ring, ◎COH]+νOH
5082	◎◎ring, ◎COH]+νOH
5041	νOH +νOH
5025	◎ring +νOH
4963	◎◎ring, ◎COH]+νOH
4906	◎◎ring, ◎COH]+νOH
4881	◎COH +νOH
4868	◎COH +νOH
4831	◎COH +νOH
4809	◎COH +νOH
4779	◎◎ring, ◎COH]+νOH
4756	◎COH◎◎νOH
4731	◎◎ring, ◎COH]◎νOH
4698	◎◎ring, ◎COH]◎νOH
4624	2νCH, 2$\nu_{as}$$CH_2$
4530	◎◎COH, ◎$_{twist}$$CH_2$]◎◎$\nu$OH
4480	◎◎ring,◎◎$_{twist}$$CH_2$]◎$\nu$OH
4451	◎◎ring,◎◎COH,◎◎$_{twist}$$CH_2$]◎$\nu$OH
4410	◎◎ring,◎◎COH,◎◎$_{twist}$$CH_2$]◎$\nu$OH
4315	◎◎ring, ◎COH]◎νCH

4270	◎◎ring, ◎COH]◎🐦CH
4227	◎◎ring, ◎COH]◎🐦CH
4178	◎◎ring, ◎COH]◎🐦CH
4089	◎◎ring, ◎COH]◎🐦CH
4068	◎◎ring, ◎COH]◎🐦CH

[a] Notation used: "2" denotes first overtones; "+" sign denotes combination transitions; ν - stretching mode; δ - deformation mode; as – antisymmetric; s – symmetric (mode).

Figures

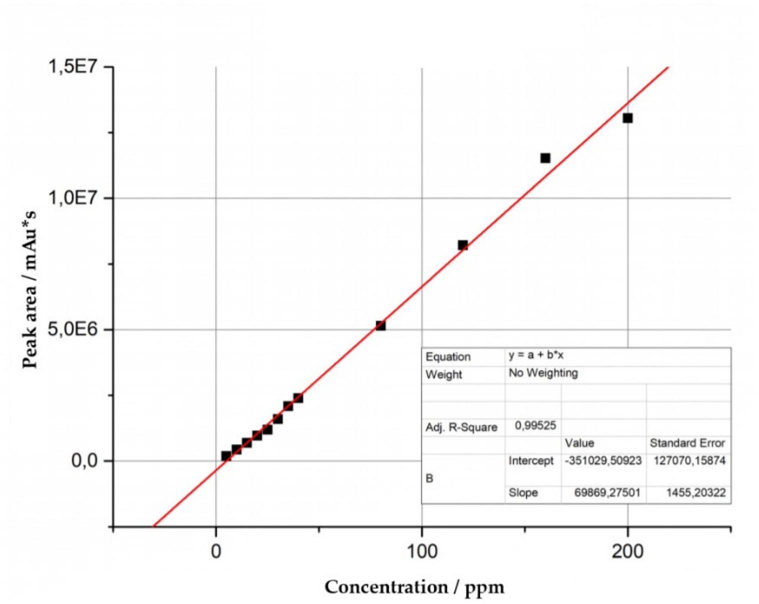

Figure S1. Calibration curve for the reference analysis (UHPLC-MWD-UHR-TOF-MS) of cyanidin-3-O-glucoside.

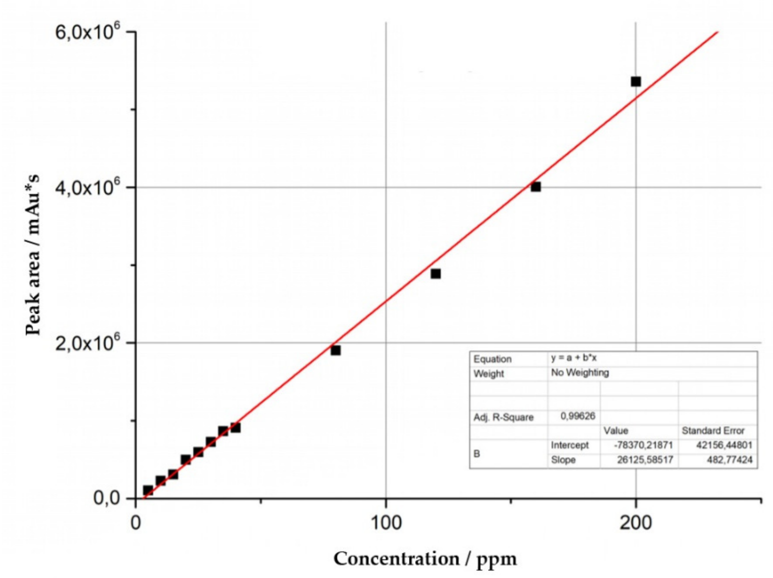

Figure S2. Calibration curve for the reference analysis (UHPLC-MWD-UHR-TOF-MS) of cyanidin-3-O-sambubioside.

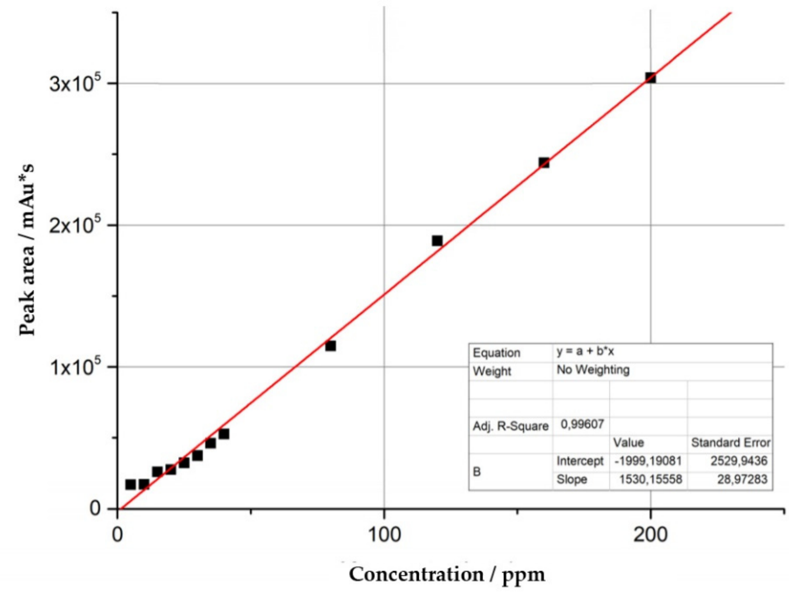

Figure S3. Calibration curve for the reference analysis (UHPLC-MWD-UHR-TOF-MS) of cyanidin-3-Osambubioside-5-glucoside.

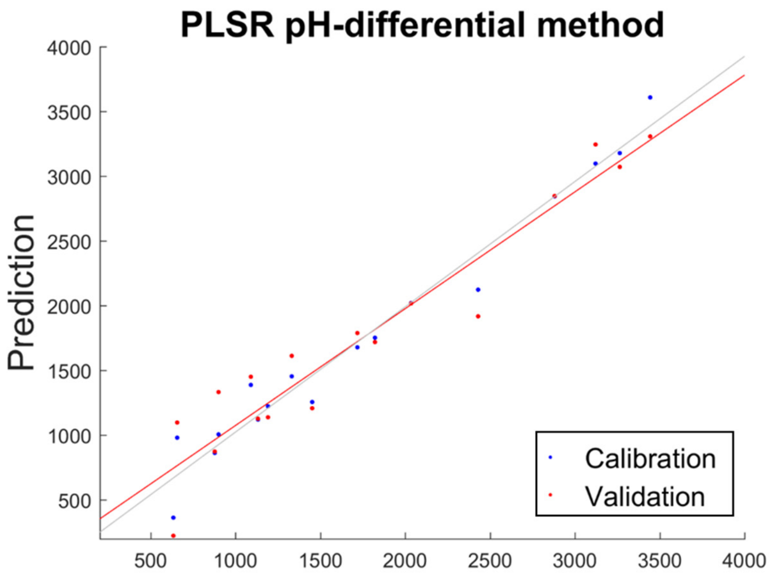

Figure S4. Prediction vs reference plot of NIR data with pH-differential reference method.

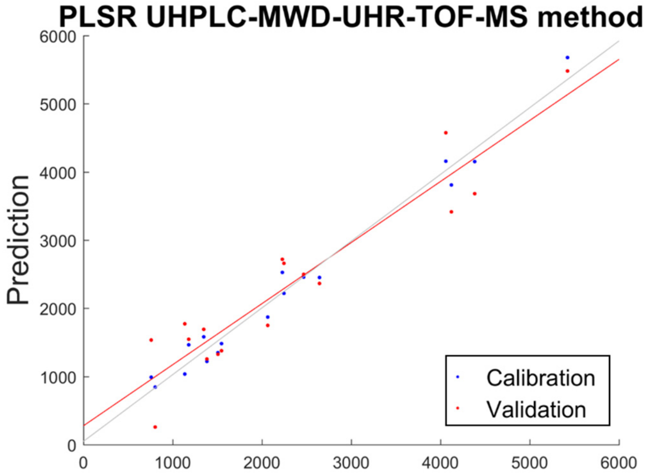

Figure S5. Prediction vs reference plot of NIR data with UHPLC-MWD-UHR-TOF-MS reference method.

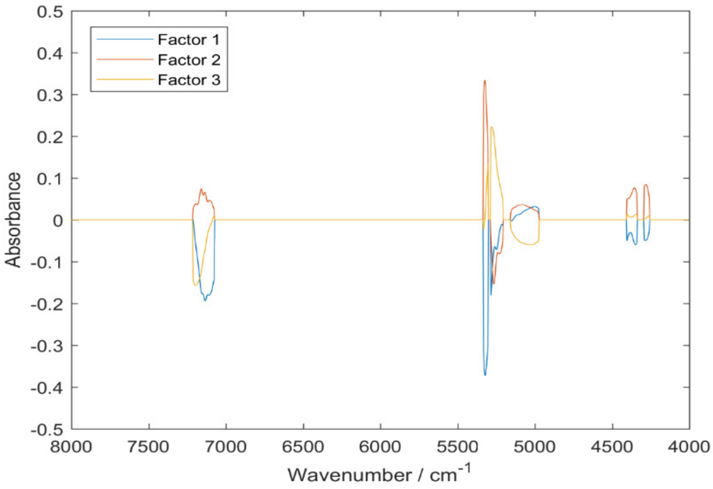

Figure S6. The loadings of the PLSR model correlating NIR spectra of sambucus fructus with pH-differential reference values for TAC content.

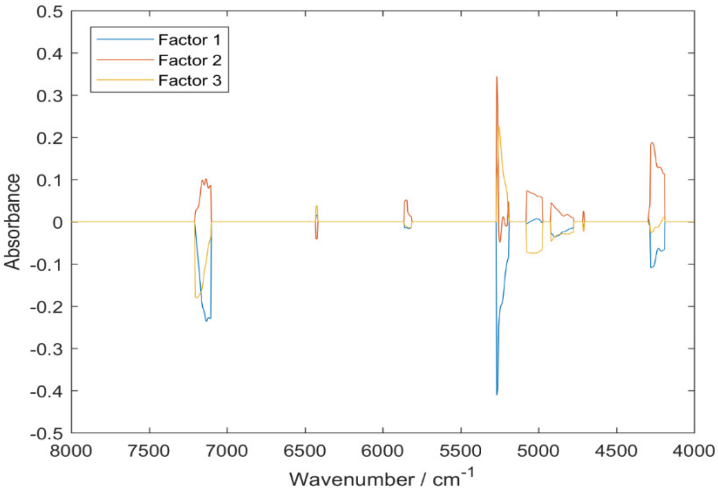

Figure S7. The loadings of the PLSR model correlating NIR spectra of sambucus fructus with UHPLC-MWD-UHR-TOF-MS reference values for TAC content.

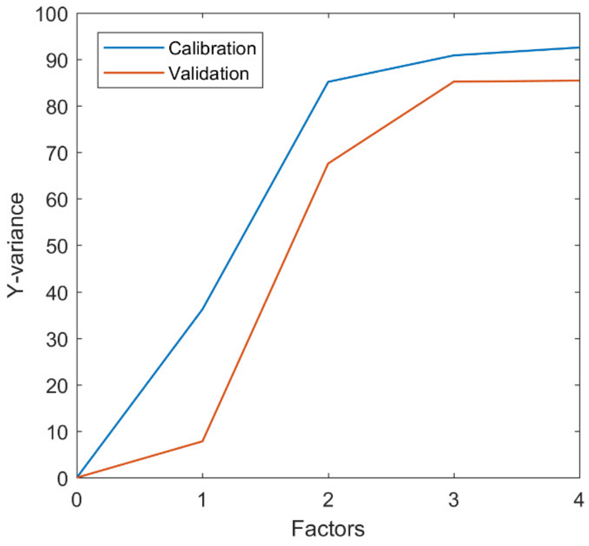

Figure S8. Cumulative explained variance plot of the PLSR model correlating NIR spectra of sambucus fructus with pH-differential reference values for TAC content.

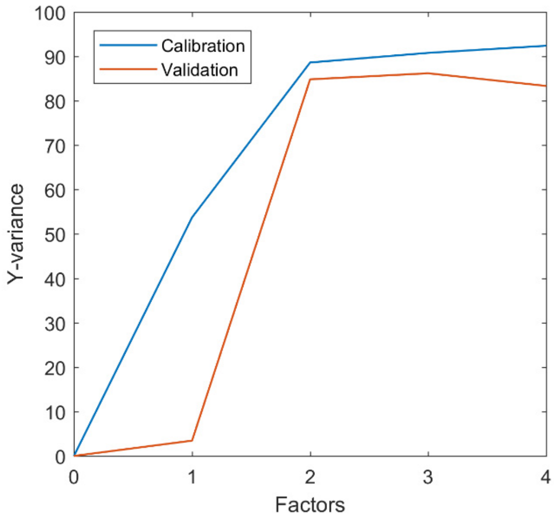

Figure S9. Cumulative explained variance plot of the PLSR model correlating NIR spectra of sambucus fructus with UHPLC-MWD-UHR-TOF-MS reference values for TAC content.

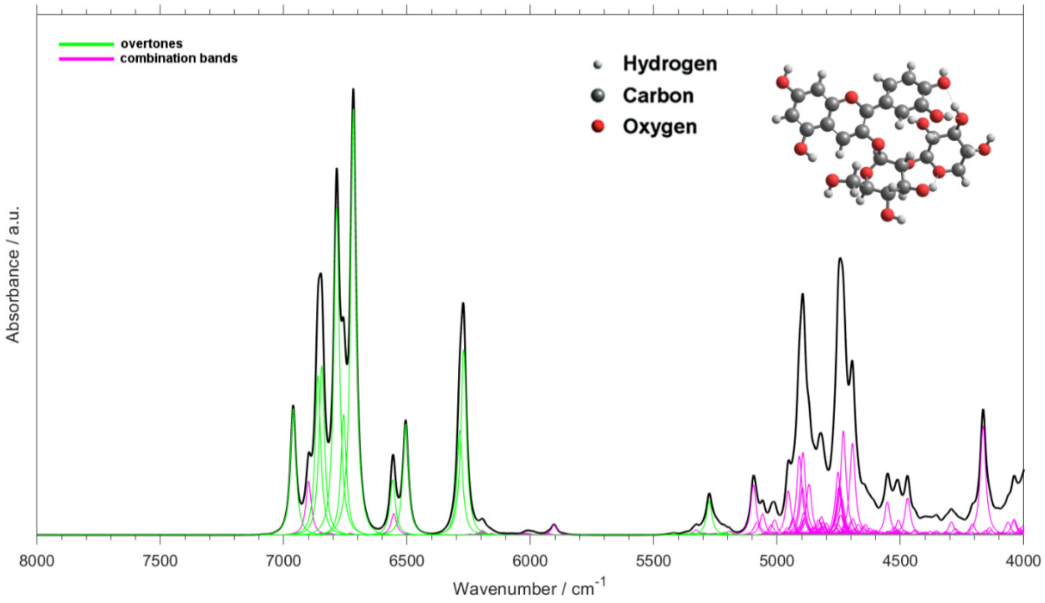

Figure S10. NIR spectrum of cyanidin-3-O-sambubioside simulated with use of quantum chemical calculations. Individual contributing overtone and combination bands are presented.

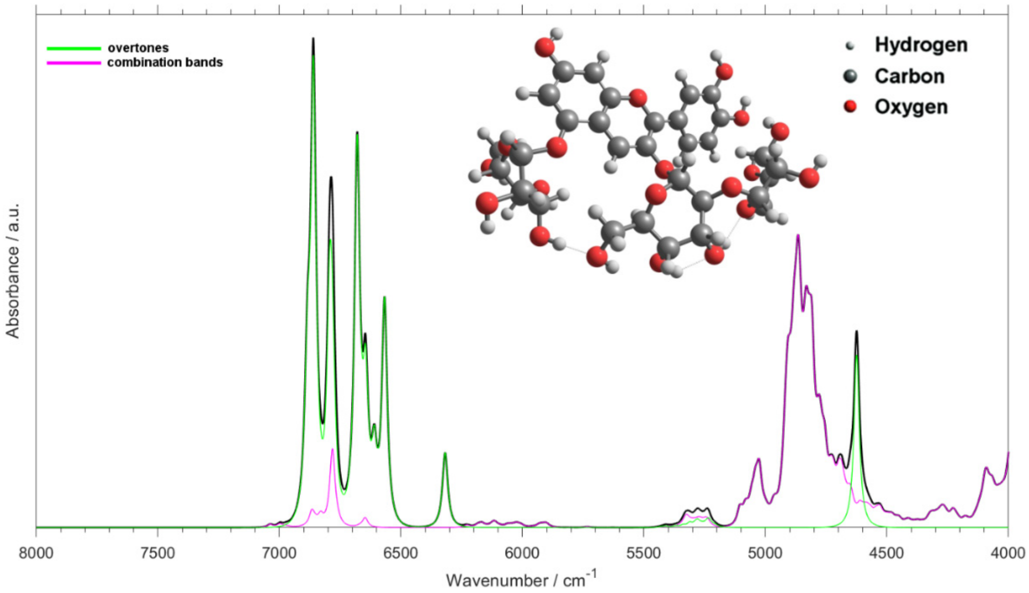

Figure S11. NIR spectrum of cyanidin-3-O-sambubioside-5-O-glucoside simulated with use of quantum chemical calculations. Summed contribution from either overtone or combination bands are presented.

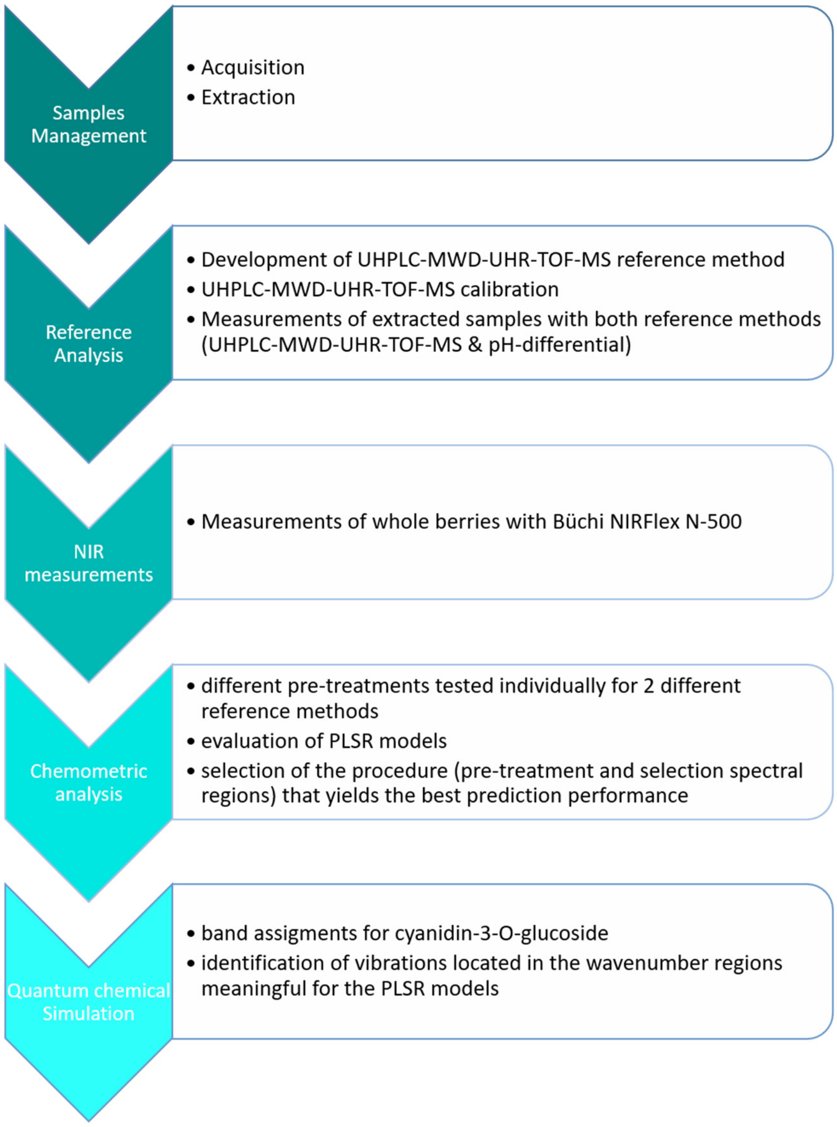

Figure S12. Workflow of the research.
